# The effects of CPAP therapy on metabolic profile and subjective sleep parameters in patients with OSA: a prospective trial study

**DOI:** 10.5935/1984-0063.20220041

**Published:** 2022

**Authors:** Zahra Banafsheh Alemohammad, Khosro Sadeghniiat-Haghighi, Mohammad Mehraban Parisa Fazlipanah, Ania Rahimi-Golkhandan

**Affiliations:** Tehran University of Medical Sciences, Occupational Sleep Research Center, Baharloo Hospital - Tehran - Iran.

**Keywords:** Sleep, Apnea, Metabolic Syndrome, Continuous Positive Airway Pressure

## Abstract

**Objective:**

Several studies confirmed a positive association between obstructive sleep apnea (OSA) and metabolic syndrome. Continuous positive airway pressure (CPAP) is the main treatment for patients with moderate and severe OSA. CPAP therapy in adults with OSA results in reduction in sleepiness, blood pressure and improvement of metabolic profile. In this study, we aimed to evaluate the effects of CPAP therapy on various components of metabolic syndrome and subjective sleep parameters in patients with OSA.

**Material and Methods:**

In this prospective trial study, 28 patients with moderate and severe OSA enrolled. Patients were asked to fill out the validated Persian version of questionnaires including Epworth sleepiness scale, insomnia severity index, STOP-BANG and Beck depression inventory - II, before and after treatment with CPAP. Weight and blood pressure were recorded before and after treatment. Only 14 patients agreed to blood sampling before and after CPAP therapy (at least 3 months of treatment). Fasting blood samples were analyzed for measuring the levels of FBS (fasting blood sugar), TG (triglyceride), total cholesterol, HDL, LDL, AST, and ALT.

**Results:**

Diastolic blood pressure, ISI and STOP-BANG score significantly decreased after treatment (*p*-value: 0.008, 0.022 and 0.004, respectively). FBS and TG levels decreased after treatment, but only TG levels had significant difference (*p*-value: 0.46 and 0.016, respectively).

**Discussion:**

CPAP therapy had positive effects on diastolic blood pressure, TG levels and ISI score. More studies with larger sample size and longer follow-up periods are warranted to investigate the effects of CPAP therapy on blood pressure, and metabolic parameters.

## INTRODUCTION

Obstructive sleep apnea (OSA) is a clinical sleep disorder characterized by complete or partial repetitive episodes of airway collapse during sleep. The prevalence of OSA estimated to be 17-24% in men and 5-9% in women. Excessive daytime sleepiness is a common symptom in patients with OSA. OSA is associated with sleep fragmentation, insomnia, intermittent hypoxia, insulin resistance, hypertension, dyslipidemia, metabolic syndrome and cardiovascular consequences^1-5^. Metabolic syndrome consists of metabolic risk factors including central obesity, hypertension, hyperglycemia, and dyslipidemia^6-8^. The prevalence of metabolic syndrome has been estimated between 20% and 40 %^9,10^. Several studies confirm a positive association between OSA and metabolic syndrome, but their causality has not been reported^11^.

Continuous positive airway pressure (CPAP) is the main treatment for patients with moderate and severe OSA. CPAP therapy in adults with OSA results in reduction in sleepiness, disease severity, blood pressure, motor vehicle accidents and improvement in quality of life^12,13^. CPAP treatment in patients with OSA results in decrease of total cholesterol and LDL levels, and increase in HDL level, but with no effect on TG levels^14^. Treating patients with moderate to severe OSA with CPAP will improve management of their hyperglycemia^15^. Previous studies showed that short-term CPAP therapy would result in reduced oxidative stress and a decrease in the severity of several parameters of metabolic syndrome such as blood pressure^16^.

OSA and metabolic syndrome have common risk factors, which are associated with an increased risk of cardiovascular consequences^17^, therefore with treatment and reduction of these risk factors, we can prevent from cardiovascular events. In this study, we aimed to evaluate the effects of CPAP therapy on various components of metabolic syndrome and subjective sleep parameters in patients with OSA.

## MATERIAL AND METHODS

### Study design

In this prospective trial interventional study, 28 patients with moderate and severe OSA enrolled. This study was conducted from 2019 to 2020, in Baharloo sleep clinic, Tehran, Iran. After first night PSG, CPAP titration performed and all of the subjects were under treatment of CPAP therapy for at least 3 months (from 3 to 12 months). Patients used the CPAP machine for >4 hours per night for at least 70% of the nights. Written consents were obtained and this study was approved by ethics committee of Tehran University of Medical Sciences (IRCT registration number: IRCT2017012832249N1).

### Study participants and measures

The patients were asked to fill out the validated Persian version of questionnaires including ESS (Epworth sleepiness scale)^18^, ISI (Insomnia severity index)^19^, STOPBANG^20^ and BDI-II (Beck depression inventory-II)^21^, before and after treatment with CPAP. Weight and blood pressure (by sphygmomanometer) in same situations were recorded before and after treatment. All of the subjects filled out the questionnaire package twice, while only 14 patients agreed to blood sampling before treatment and 3 months following beginning of the treatment. Fasting blood samples were analyzed for measuring the levels of FBS (fasting blood sugar) (by glucose oxidase assay method), TG (triglyceride) (by lipase peroxidase colorometric method), total cholesterol, HDL (high-density lipoprotein) and LDL (low-density lipoprotein) (by direct enzymatic colorimetric method), AST (aspartate aminotransferase) and ALT (alanine aminotransferase, by NADH Kinetic UV method). Inclusion criteria consist of adult patients with OSA and clinical indication for PAP therapy. Exclusion criteria consist of pregnancy, cardiopulmonary disorder, upper airway cancer or surgery, change or new administration of drug for hyperlipidemia, hypertension and diabetes mellitus. Time of CPAP usage less than 4h and 70% of the days was another exclusion criterion.

### Polysomnography (PSG)

PSG is the gold standard test for diagnosing of OSA. Electroencephalography, electrooculography, electrocardiography and electromyography performed during overnight test (PSG device: N7000 EMBLA Natus Company, USA). Apnea events are defined when airflow reduction is 90% and higher from baseline for at least 10 seconds. Hypopnea events are defined when airflow reduction is 30% and higher from baseline for at least 10 seconds, and are associated with arousals or oxygen saturation reduction >3%^22^. AHI (apnea-hypopnea index) equal or greater than 15 and 30 were categorized as moderate and severe OSA, respectively. Analysis of PSGs was performed according to AASM 2013 guideline by the same sleep medicine specialist for all subjects^22^.

### CPAP titration

A second night PSG for titration of CPAP pressure according to AASM guideline was performed for the patients who were recommended to use CPAP device for treatment of OSA^22^. CPAP push the air with positive pressure through an interface mask and this positive airway pressure will prevent from frequent collapse of upper airways in patients with OSA^23^. Optimal pressure for CPAP therapy was obtained by the same sleep medicine specialist for all patients.

### ESS questionnaire

ESS is a self-reported questionnaire for evaluating daytime sleepiness in different situations. This scale consists of eight questions, with score from 0 to 3 for each item. Total score ranges from 0 to 24. Clinically significant daytime sleepiness was defined in scores equal or greater than 10^18^.

### ISI questionnaire

ISI is a self-administrated scale for assessing severity of insomnia over the past month. This questionnaire consists seven items with summed score ranging from zero to 28. Total scores from 8 to 14 and equal or greater than 14 interpreted as sub threshold and clinically significant insomnia, respectively^19^.

### STOP-BANG questionnaire

The STOP-BANG questionnaire consists of two parts: STOP part including snoring, daytime tiredness, breathing stop during sleep, which observed by another individual, and having or be treated for hypertension; and BANG part including body mass index (BMI), age, neck circumference, and gender. BMI more than 35kg/m^2^, age more than 50 years, neck circumference more than 40cm, and male gender categorized as positive scores. Patients with positive answer to three or more of eight items labeled as high risk for OSA^20^.

### BDI-II questionnaire

BDI-II is a self-reported questionnaire for evaluating the severity of depression symptoms. This scale consists of 21 questions with the 4-point Likert scale ranging from zero to three, which reflects participants feeling over the past two weeks. Total score ranges from zero to 63 with higher scores representing greater depressive mood^21^.

### Statistical analysis

All measurements are expressed as mean ± standard deviation. Mann-Whitney U test was used for comparing continuous variables before and after CPAP treatment. All *p*-values were two-tailed, and *p*-value lesser than 0.05 was considered statistically significant. Predictive analytics software (PASW) version 18 was used for the statistical analysis.

## RESULTS

Of 28 subjects, 17 (60%) were female. The mean age and BMI of participants was 51.2±10.9 and 30.9±5.5, respectively. The mean AHI of patients before treatment with CPAP was 57±25.6 when after CPAP therapy, mean AHI was reduced to 2.4 (*p*-value<0.0001).

Systolic and diastolic blood pressure, weight, ESS, ISI, STOP-BANG, and BDI of the subjects before and after CPAP therapy are shown in Table 1. Systolic blood pressure decreased from 124.4±14mmHg to 116.3±13.9mmHg but there was no significant difference, whereas there was significant decrease in diastolic blood pressure (from 84.6±7.7 to 75.3±11.1). ESS scores before and after treatment reported 9.3±5.7 and 9.3±5, respectively, whereas ISI score decreased from 14.4±7.5 to 11.5±6.7. In current study, diastolic blood pressure, ISI and STOP-BANG score significantly decreased after treatment (*p*-value: 0.008, 0.022 and 0.004, respectively).

**Table 1:** Physical examination and subjective sleep parameters before and after CPAP therapy.

	Before CPAP therapy	After CPAP therapy	*p*-value
Systolic blood pressure	124.4±14	116.3±13.9	0.08
Diastolic blood pressure	84.6±7.7	75.3±11.1	0.008
Weight	87.6±16.4	87.8±15.9	0.79
ESS	9.3±5.7	9.3±5	0.97
ISI	14.4±7.5	11.5±6.7	0.022
STOP-BANG	4.6±1.1	3.9±1.6	0.004
BDI	20.1±16.7	19.1±18.4	0.75

For evaluating metabolic profile and liver function tests (LFT), only 14 subjects agreed to give twice blood samples (which are shown in Table 2). FBS levels decreased from 111.4±25.8 to 108.1±18.3 without significant difference, whereas TG levels before and after treatment significantly decreased (170.3±49.9 and 151.8±53.2, respectively). Total-cholesterol levels showed no difference before and after CPAP therapy (176.3±39.1 and 176.9±46.1, respectively). In this study, FBS and TG levels decreased after treatment, but only TG levels had significant difference (*p-*value: 0.46 and 0.016, respectively).

**Table 2:** Metabolic profile and LFT before and after CPAP therapy.

	Before CPAP therapy	After CPAP therapy	*p*-value
FBS	111.4±25.8	108.1±18.3	0.46
TG	170.3±49.9	151.8±53.2	0.016
Total-cholesterol	176.3±39.1	176.9±46.1	0.97
HDL	44.6±9.3	39.1±13.9	0.13
AST	24.8±7.2	24.4±9.1	0.90
ALT	28.3±14.8	28.5±12.8	0.97

## DISCUSSION

In current study, diastolic blood pressure, ISI and STOPBANG score significantly decreased after treatment (*p*-value: 0.008, 0.022 and 0.004, respectively). FBS and TG levels decreased after treatment, but only TG levels had significant difference (*p*-value: 0.46 and 0.016, respectively).

Wilcox et al., in 1998^24^, for the metabolic syndrome (syndrome X) combined with OSA proposed the name syndrome Z to affirm the close association of components of the metabolic syndrome with OSA.

In this present study, CPAP therapy has been led to significant decrease in diastolic blood pressure (9.3mmHg, *p*-value: 0.08), systolic blood pressure also decreased after treatment but the difference was not significant (8.1 mmHg, *p*-value: 0.08). This is probably due to the effect of CPAP in prevention of episodic hypoxemia and nocturnal sympathetic activation. In a study of 194 patients, were randomly assigned to receive CPAP, the CPAP group achieved a greater decrease in 24-hour mean blood pressure and diastolic blood pressure, but not in 24 hour systolic blood pressure compared with the control group. Furthermore, in the CPAP group greater percentage of patients displaying a nocturnal blood pressure dipper pattern at the 12-week follow-up than in the control group^25^. Another study on 35 OSA patient indicated that CPAP therapy led to decrease of 9.76 in systolic blood pressure and 3.49mmHg in diastolic blood pressures^26^. More studies with larger sample size and longer follow-up periods are warranted to investigate the effects of CPAP therapy on systolic and nocturnal blood pressure.

Effective CPAP therapy may lead to weight loss by several proposed mechanisms, including increased physical activity and increased responsiveness to leptin. In a study by Redenius et al., in 2008^27^, BMI was recorded at the time of diagnosis and at follow up approximately 1 year (10-14 months) later. In this study, BMI of treatment and control subjects did not significantly differ. BMI did not significantly decrease in any group treated with CPAP. Perhaps greater CPAP adherence leads to a greater decrease in leptin and resultant increased caloric intake^27^. In current study, there was no significant difference between patients’ weight and BMI after treatment. This result may be due to limited sample size and shorter follow-up period.

In obesity energy intake exceeds energy expenditure. In normal individuals, energy expenditure typically decreases during sleep. In individuals with sleep apnea, energy expenditure in sleep increases during apneic sleep and decreases with CPAP therapy^28^.

In current study, no difference between patients’ weight before and after CPAP therapy was reported. This may be due to short-term treatment (3-12 months).

In our study, for evaluating metabolic profile and liver function tests (LFT), only 14 subjects agreed to give twice blood samples. FBS and TG levels decreased after treatment, but only TG levels had significant difference (*p*-value: 0.46 and 0.016, respectively). Reports about the effect of CPAP on lipid profile are controversial. In a study included 35 OSA patients results indicated that CPAP therapy led to decrease LDL to 6.27mg/ dl and increase HDL to 0.75mg/dl (*p*<0.001) with treatment. The changes of FBS, TG, and cholesterol were not significant (*p*>0.05)^26^.

In another study on patients with moderate-to-severe obstructive sleep apnea, CPAP therapy improve insulin secretion capacity; reduce total cholesterol, low-density lipoprotein and leptin levels. Leptin showed considerable relationship with insulin resistance and this relationship remained after 8 weeks of CPAP therapy^28^.

In another study of 94 patients with moderate-tosevere OSAS under CPAP treatment, plasma ALT and AST were measured before and after 4 weeks of CPAP, four weeks of CPAP treatment has no beneficial effect on ALT and AST levels when compared to sub- therapeutic CPAP in patients with OSAS. Therefore, CPAP does not seem to improve biochemical markers of potential nonalcoholic fatty liver disease in OSAS patients^29^.

The mean ESS scores in our study were not decreased after treatment with CPAP. In another study, mean ESS scores among moderate and severe OSA patients improved significantly after three months of CPAP therapy^4^.

In another study of 92 subjects, mean ESS score was 11.31±5.6 at baseline vs. 6.9±3.3 after 1 month CPAP therapy (*p*=0.02)^30^. In our study daytime sleepiness was not a significant complaint among participants (mean ESS score was lesser than 10), the mean ESS scores before and after treatment in our sample were 9.3±5.7 and 9.3±5, respectively (*p-*value: 0.97), which shows our participants were not sleepy at the base, not changed after CPAP therapy.

In our study, ISI scores after CPAP therapy decreased significantly (14.4±7.5 versus 11.5±6.7, *p*-value: 0.022). In our sample female subjects were more than male ones (66% female). Females with OSA with lower AHI appear to be more symptomatic than males and they report insomnia more frequently as a symptom of OSAS^31^.

In our study the mean BDI score decreased just one point (from 20.1±16.7 to 19.1±18.4, *p*-value: 0.75). The results of another study suggest that BDI score decreased from 19.7 to 10.8 after 2 months of CPAP treatment (*p*<0.01). Patients with major depression with residual depressive symptoms after pharmacotherapy and symptoms of suspected OSA, such as witnessed apnea, loud snoring, obesity, and excessive daytime sleepiness, should be evaluated for sleep apnea by polysomnography and treated with CPAP. CPAP treatment may significantly improve residual depressive symptoms by improvement of daytime sleepiness in these patients^32^.

In our study, STOP-BANG score significantly decreased after treatment, obvious snoring and daytime sleepiness and observed apnea resolved after treatment and blood pressure can control more easily.

### Limitations

Because of low adherence to CPAP therapy, expensive treatment, and COVID-19 pandemic crisis, limited sample size was a limitation. More studies with larger sample size and longer follow-ups are recommended. The large variability of the follow-up time (3-12m) is a possible reason for this paper’s results. The impossibility to use ambulatory blood pressure monitoring as a tool to see blood pressure changes was another limitation in this study.

## CONCLUSION

CPAP therapy had positive effects on diastolic blood pressure, TG levels and ISI score in our study. Diastolic blood pressure, ISI and STOP-BANG score significantly decreased after treatment. FBS and TG levels decreased after treatment, but only TG levels had significant difference.
